# Direct and indirect single electron transfer (SET)-photochemical approaches for the preparation of novel phthalimide and naphthalimide-based lariat-type crown ethers

**DOI:** 10.3762/bjoc.10.47

**Published:** 2014-02-27

**Authors:** Dae Won Cho, Patrick S Mariano, Ung Chan Yoon

**Affiliations:** 1 Department of Chemistry, Yeungnam University, Gyeongsan, Gyeongbuk 712-749, Korea; 2 Department of Chemistry and Chemical Biology, University of New Mexico, Albuquerque, NM 87131, USA; 3 Department of Chemistry and Chemistry Institute of Functional Materials, Pusan National University, Busan 609-735, Korea

**Keywords:** lariat-type crown ethers, SET-promoted photocyclization, α-silylether-terminated imides

## Abstract

In this review, we describe direct and indirect photochemical approaches that have been developed for the preparation of phthalimide- and naphthalimide-based, lariat-type crown ethers. The direct route utilizes a strategy in which nitrogen-linked side chains containing polyethoxy-tethered phthalimides and naphthalimides, possessing terminal α-trialkylsilyl groups, are synthesized utilizing concise routes and UV-irradiation to form macrocyclic ring systems. In contrast, the indirect route developed for the synthesis of lariat-type crown ethers employs sequences in which SET-promoted macrocyclization reactions of α-trialkylsilyl-terminated, polyethoxy-tethered phthalimides and naphthalimides are followed by a side chain introduction through substitution reactions at the amidol centers in the macrocyclic ethers. The combined observations made in these investigations demonstrate the unique features of SET-promoted photocyclization reactions that make them well-suited for the use in the synthesis of functionalized crown ethers. In addition, while some limitations exist for the general use of SET-photochemical reactions in large-scale organic synthesis, important characteristics of the photoinduced macrocyclization reactions make them applicable to unique situations in which high temporal and spatial control is required.

## Review

Since the 1970s, studies by a number of organic photochemists have demonstrated that a large number of unique single electron transfer (SET) mechanistic pathways are followed in photochemical reactions [1–10]. Owing to its potentially large energetic driving force, SET to or from singlet and triplet excited states often plays a key role in guiding the nature of photochemical processes. Generally, photoinduced SET from electron donors to acceptors results in the generation of highly reactive ion radical pairs, which participate in facile and selective secondary reactions as a part of pathways leading to product formation. Thus, photochemical reactions promoted by excited state SET are governed by the chemical properties of charged and neutral radical intermediates [11–17].

**Scheme 1:**
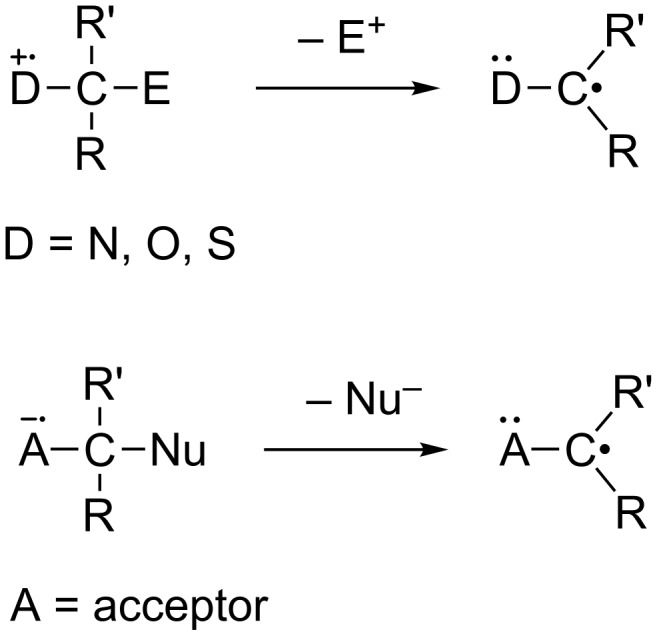
α-Heterolytic cleavage in ion radicals.

A common pathway followed in reactions of ion radicals involves an α-heterolytic fragmentation in which either an electrofugal (E^+^) or nucleofugal (Nu^−^) group is transferred from a position adjacent to respective positively and negatively charged radical centers (Scheme 1). These fragmentation reactions produce neutral radicals, which then participate in typical radical reactions, including C–C bond formation and addition to unsaturated centers. Because of their importance, α-heterolytic fragmentation reactions of radical anions and cations have been intensively studied from synthetic and mechanistic perspectives [11–34].

**Scheme 2:**
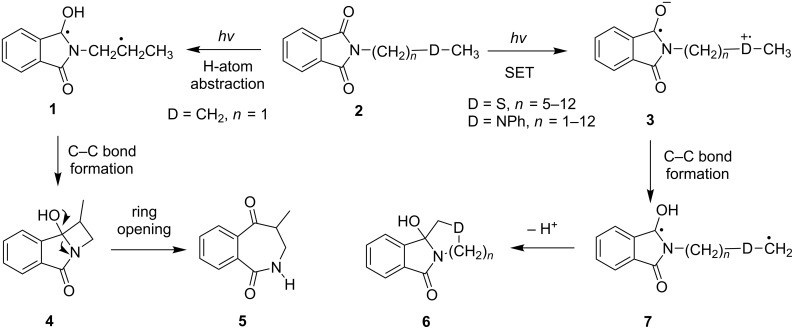
Photochemical reaction pathways of *N*-alkylphthalimides.

In intensive early investigations, Kanaoka [35–40] and Colye [41–44] demonstrated the participation of phthalimides in several different kinds of interesting SET-promoted photochemical reactions. Since phthalimides have high excited state reduction potentials (^1^E_1_(–) = 2.3 V; ^3^E_1_(–) = 1.6 V), their excited states take part in efficient SET-promoted pathways when coupled with both n- and π-electron donors that have oxidation potentials less than ca. 2.3 V. While photoirradiation of *N*-alkylphthalimides **1** brings about typical H-atom abstraction processes (e.g., Norrish type II reaction) [38,45–46] that produce cyclic amides **5** via **4** (Scheme 2), photoirradiation of phthalimides containing thioether and/or amine chains **2** promotes more rapidly the intramolecular SET from the heteroatom donors (S and N) to the phthalimide excited states. The SET processes form zwitterionic biradical intermediates **3**, in which a proton transfer and an α-heterolytic fragmentation proceeds, produce biradicals **7** that are precursors of the heterocyclic products **6** (Scheme 2).

**Scheme 3:**
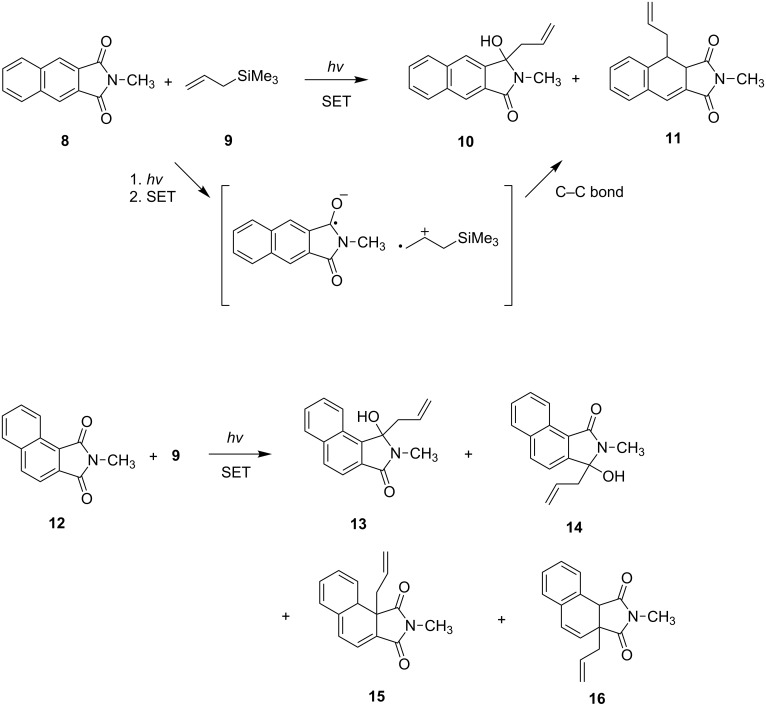
Photoreactions of *N*-methylnaphthalimides **8** and **12** with allylsilane **9**.

Photochemical reactions of naphthalimides with electron donors have also been intensively studied. For example, Kubo and coworkers [47] showed that photoreactions of *N*-methyl-1,2- and 2,3-naphthalimides **8** and **12** with allylsilane **9** in MeCN can produce allylation products [48–52] that arise by a well-known sequence involving intermolecular SET, radical cation desilylation and radical coupling (Scheme 3).

Allyl- and enolsilanes serve as important substrates in numerous synthetically useful ground and excited state reactions [53–54]. The most common roles of these substances are to act as the respective equivalents of allylic anions and enolate ions. When coupled with strong electrophiles, allylsilanes and enolsilanes undergo addition reactions in which the α-trialkylsilyl groups are readily transfered even to weak silophiles.

**Scheme 4:**
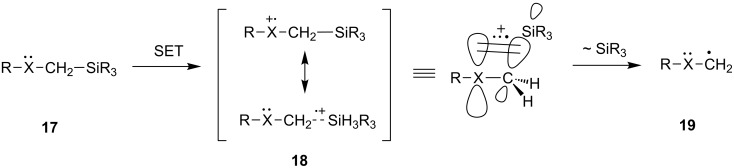
Regioselective generation of carbon-centered free radicals through sequential SET-desilylation processes.

Over the past two decades, a variety of photochemical and electrochemical investigations have uncovered another interesting reactivity profile of organosilanes. Among a number of n-electron donors, those that contain α-trialkylsilyl substituents **17** have been observed to undergo ready SET oxidation to generate silicon stabilized cation radicals **18** (Scheme 4). Studies by Yoshida and coworkers [55–61] have shown that α-silyl cation radicals **18** are stabilized by an hyperconjugation-type overlap of high energy σ_C-Si_ orbitals with half-filled *p*-orbitals on the donor atoms. This feature causes α-trialkylsilyl-substituted electron donors to have comparable low oxidation potentials. In addition, the odd electron and positive charge delocalization arising by the orbital overlap, lowers the σ_C-Si_ bond dissociation energy and makes the silicon center more electropositive. Consequently, short-lived α-silyl cation radicals typically undergo fast, silophile-promoted desilylation to form the carbon centered free radicals **19** (Scheme 4). Based on the low oxidation potentials and known reactivity profile of cation radicals arising from α-trialkylsilyl substituted n-electron donors, it is feasible to design substrates that undergo sequential SET-desilylation processes to produce radicals or biradical intermediates in a highly regioselective and efficient fashion.

**Scheme 5:**
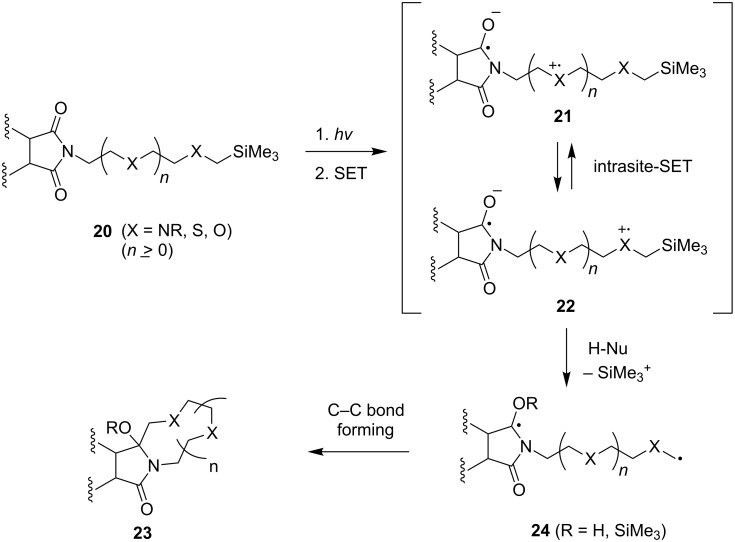
Mechanistic pathway of photochemical reactions of α-silyl n-electron donor-linked imides systems.

Previous studies [31–34,62–67] in our laboratories resulted in the development, mechanistic elucidation, and synthetic application of various kinds of SET-promoted photocyclization reactions of α-trialkylsilyl donor-linked imide acceptor systems and led to an understanding of the factors controling the chemical selectivities and efficiencies. For example, we have demonstrated that intramolecular SET-photochemical reactions of linked α-trimethylsilyl n-electron donor-phthalimides/naphthalimides produce functionalized macrocyclic poly-ethers, -thioethers, -amides, and -peptides via the intermediacy of interconverting zwitterionic biradicals **21** and **22**. The biradicals **24** generated by silyl transfer from the zwitterionic biradicals undergo C–C bond formation to form macrocyclic products **23** (Scheme 5). In addition, these studies have shown that the length and nature of the chain linking the α-trimethylsilyl n-electron donor centers and the arylimide acceptors play important roles in controlling the rate of formation of the zwitterionic biradicals **21** and **22** and, thus, affecting the yield of the macrocyclic products [31,33–34,65–67]. Importantly, these efforts provided a solid foundation for the design of new strategies for the preparation of interesting members of the crown ether family.

Substances that possess polyheteroatom-containing macrocyclic rings were carefully investigated owing to their interesting chemical and biological properties. Prime examples of these compounds are crown ethers, whose preparation, and metal and ammonium cation-binding properties were explored thoroughly by Petersen and Lehn [68–69]. Early pioneering efforts by Gokel and coworkers [70–73] in this area have shown that novel, lariat-type crown ethers display unique binding properties with a variety of cationic guests. In typical cases, carbon or nitrogen-pivot lariat crown ethers are comprised of crown ether cores and one or more heteroatom-containing side arms that are covalently bonded to carbon or nitrogen in the core. The skeletal framework and position of electron donor sites in these substances are ideally suited to capture cations in the form of chelate structures [70–79].

Several practical synthetic protocols have been developed to prepare members of the crown ether familiy. In most cases, the approaches utilize the cyclization of substrates that contain electrophilic and nucleophilic centers at the opposite ends of a poly-heteroatom-containing chain. The features of these types of reactions dictate that high dilution conditions should be employed in order to maximize the cyclization to suppress the polymerization. In some cases, methods that rely on the preorganization of linear precursors have used to improve the efficiency of crown ether-forming reactions [80–81]. Owing to the continuing importance of members of the crown ether family, new methods for their synthesis are still in demand.

**Scheme 6:**
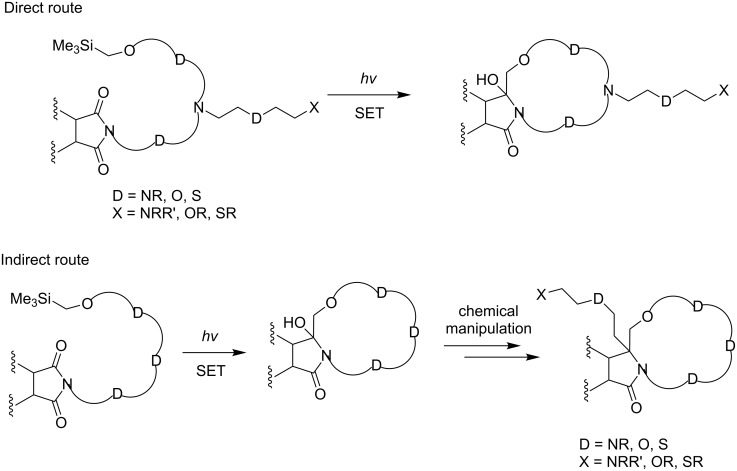
Direct and indirect photochemical approaches for the preparation of lariat-type crown ethers.

In the current review, direct and indirect photochemical approaches that we have devised for the preparation of imide- (e.g., phthalimide and naphthalimide) derived lariat-type crown ethers are described. The direct route utilizes a strategy in which nitrogen-linked side chains containing polyethoxy-tethered phthalimides and naphthalimides, possessing terminal α-trialkylsilyl groups, are synthesized utilizing concise routes and UV-irradiation to form the macrocyclic ring systems (Scheme 6). In contrast, the indirect route developed for the synthesis of lariat-type crown ethers employs sequences in which the SET-promoted macrocyclization reactions of the α-trialkylsilyl-terminated, polyethoxy-tethered phthalimides and naphthalimides are followed by a side chain introduction through substitution reactions at the amidol centers in the macrocyclic ethers (Scheme 6).

### Direct approaches for the preparation of imide-derived lariat-type crown ethers

**Scheme 7:**
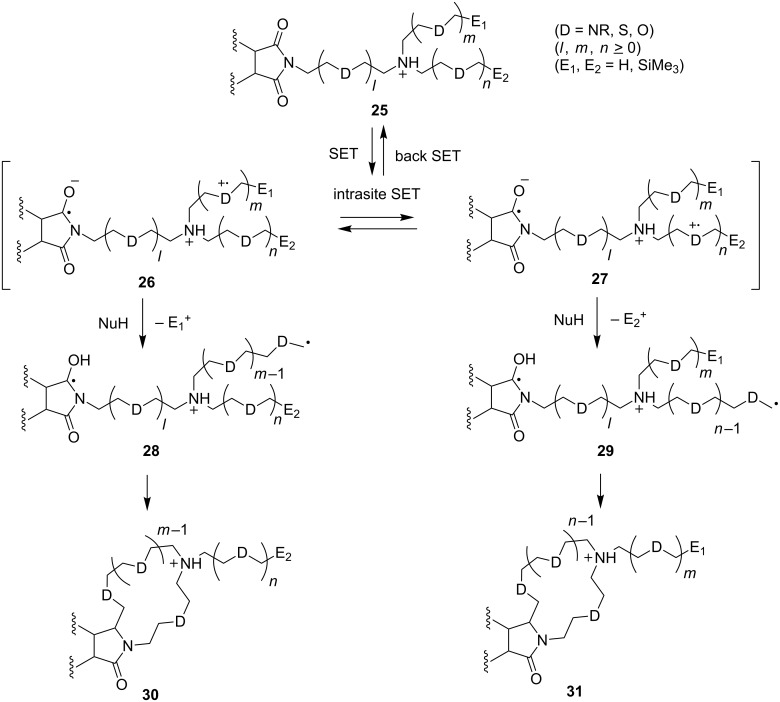
Feasible mechanistic pathways of photochemical reactions of donor atom-linked phthalimides and 2,3-naphthalimides.

Classical synthetic strategies for the preparation of lariat-type crown ethers have utilized ground state polar cyclization reactions between electrophiles/nucleophiles that require low concentrations of reactants in order to minimize competing, undesirable intermolecular reactions including polymerization reactions. In earlier studies, we explored a number of excited states of photochemical reactions of phthalimides and 2,3-naphthalimides **25**, which contain a variety of *N*-linked donor atoms (Scheme 7). In these processes, an intramolecular SET from α-silyl ether donors to excited states of the imide acceptors occurs to generate rapidly interconverting zwitterionic radicals **26** and **27**, each of which can undergo secondary reactions to generate biradical intermediates **28** and **29** that serve as precursors of cyclic products **30** and **31**.

The results of exploratory studies revealed that the chemical yields and regiochemical selectivities of these photochemical reactions are governed by several factors. In case where the rates of the intrasite-SET are low compared to those of cation radical fragmentation reactions (*k*_E1_ or *k*_E2_), photoproduct ratios are governed by the relative rates of (1) SET from the respective donor sites in the excited state of the substrate, and (2) α-fragmentation vs back-SET. In another case, where the intrasite-SET is more rapid than α-fragmentation at the cation radical centers, an equilibrium is attained between the zwitterionic biradicals, the mol fraction of each being controlled by the redox potential of each donor site. Moreover, in these cases, the product yields will rely on the relative rates of the competing fragmentation reactions (*k*_E1_ vs *k*_E2_) as a result of the fact that the energy barriers for these processes are higher than that of intrasite-SET [31,62–67]. As a result, the number, location, types, and reactivity of zwitterionic radical centers arisen by either direct or intrasite-SET will influence the efficiencies of redox reactions of polydonor-imide acceptor systems.

### Direct approach. SET-promoted photochemical reactions of branched chain-tethered phthalimides

**Scheme 8:**
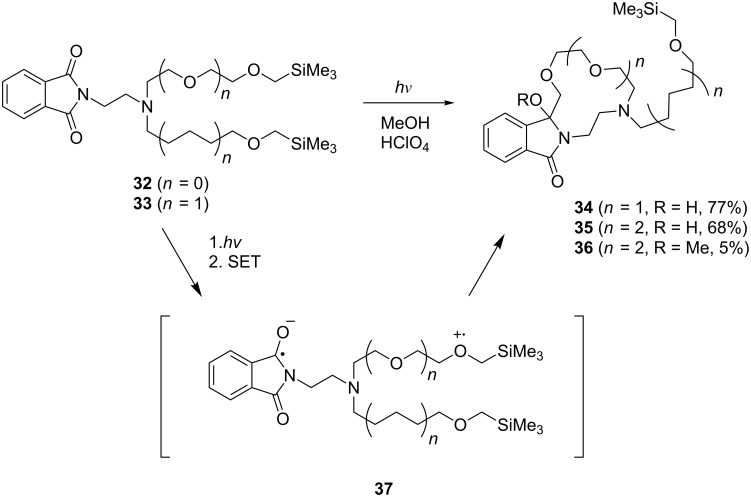
Photoreactions of branched, bis(α-silylether)-terminated phthalimides.

**Scheme 9:**
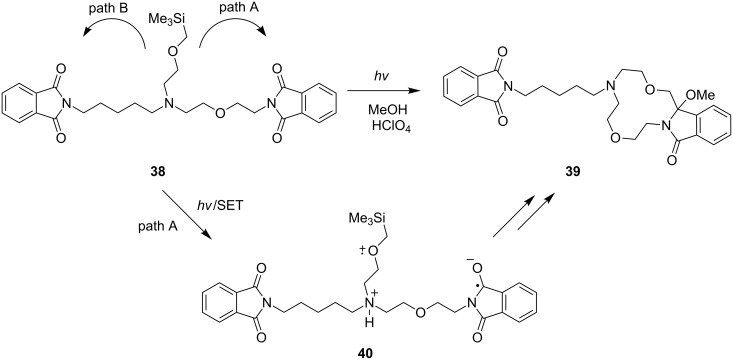
Photoreactions of the α-silylether-linked bisphthalimide acceptor.

In order to explore the feasibility of using the direct approach for the preparation of lariat-type crown ethers, branched, bis(α-silyl ether)-terminated chains containing phthalimides **32** and **33** were prepared and subjected to UV-irradiation in MeOH solution containing HClO_4_ (Scheme 8) [33]. Acidic photoreaction mixtures were used for the photochemical processes to cause protonation and prevent undesirable SET from nitrogen donor sites in the side chains. At the outset, we expected that photoirradiation of the polyethylenoxy- and polymethylene-linked phthalimides **32** and **33** would bring about a competitive formation of zwitterionic biradicals having cation radical centers located at the α-silyl ether donor sites which are located nearly equidistant from the excited phthalimide acceptor. In addition, sequential desilylation and radical coupling of each of these intermediates would lead to the formation of two types of lariat-type azacrown ether products. However, the experimental results showed that photoreactions of both **32** and **33** occur cleanly to form the lariat-type crown ethers **34** and **35** or **36** predominantly or exclusively. This observation suggests that SET through the polyethyleneoxy chains is more efficient than that through the corresponding polymethylene chains. As a result, zwitterionic biradicals like **37** (Scheme 8) are more efficiently generated in these processes, leading to the generation of the more oxygen atoms-containing lariat-type azacrown ethers **34** and **35** or **36**. A similar factor operates to control the regioselectivity of the photochemical reaction of the bis-acceptor tethered α-silyl ether **38** (Scheme 9) [65]. In this case, photoirradiation of an acidic methanol solution results in exclusive formation of **39** via a mechanistic route initiated by selective SET-desilylation from the α-silyl ether donor site linked via the polyethyleneoxy chain (path A). The combined results showed that the regioselectivity of SET-promoted cyclization reactions leading to macrocyclic amidols, is highly controlled by the nature of the chain linking the α-silyl ether donor to the phthalimide moiety, an important feature in terms of the use of this approach for efficient synthesis of lariat-type azacrown ethers.

**Scheme 10:**
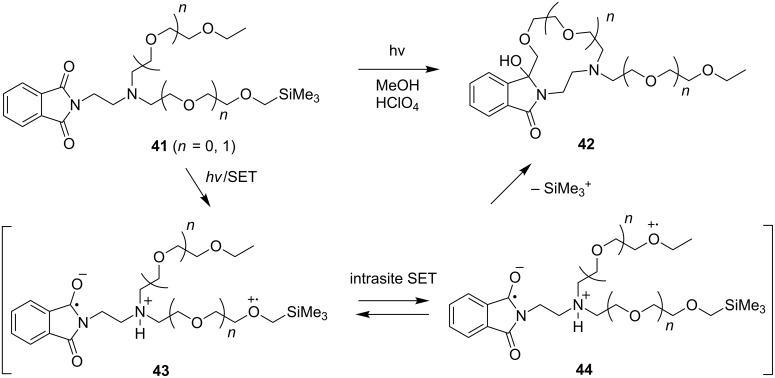
Photoreactions of branched, silyl- and non-silyl-polyethylenoxy-linked phthalimides.

To investigate routes for the preparation of lariat-type azacrown ethers that contain side chains possessing more than one heteroatom, the branched, silyl and non-silyl ether such as polyethylenoxy-linked phthalimides **41** were prepared and subjected to photochemical studies (Scheme 10) [33]. The interconverting zwitterionic radicals **43** and **44** formed by excited state SET in these systems are capable of undergoing respective desilylation or α-CH deprotonation to form the corresponding biradical intermediates. Because the cation radical desilylation is a much faster process than the deprotonation, we anticipated that these photoreactions would produce the lariat-type azacrown ethers **42** predominantly. As expected, photoreactions of **41** in acidic methanol solution take place with high efficiencies and regioselectivities to form the respective lariat-type crown ether products **42**.

**Scheme 11:**
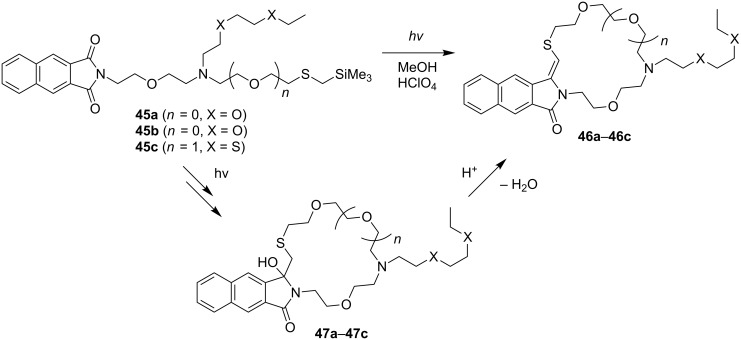
Photoreactions of branched, non-silyl ether and silyl-thioether-terminated naphthalimides.

In a similar manner, naphthalimides **45a**–**c** containing non-silyl ether and thioether, and silyl-thioether-terminated, *N*-branched side chains undergo photoreactions to generate the corresponding polyether side chain containing the lariat-type azacrown ethers **47a**–**c** exclusively (Scheme 11). As observed in earlier studies [34], the initially formed photoproducts undergo in situ dehydration to produce the vinyl sulfide products **46a**–**c** [82].

The photoreactions shown in Scheme 10 and Scheme 11 demonstrate that the zwitterionic biradical intermediates generated in these reactions by photoinduced intramolecular SET undergo highly chemoselective SET-desilylation reactions. As such, this type of reactivity profile can be incorporated into the synthesis for the preparation of a wide variety of azacrown ethers that possess different types of polyheteroatom containing side chains.

**Scheme 12:**
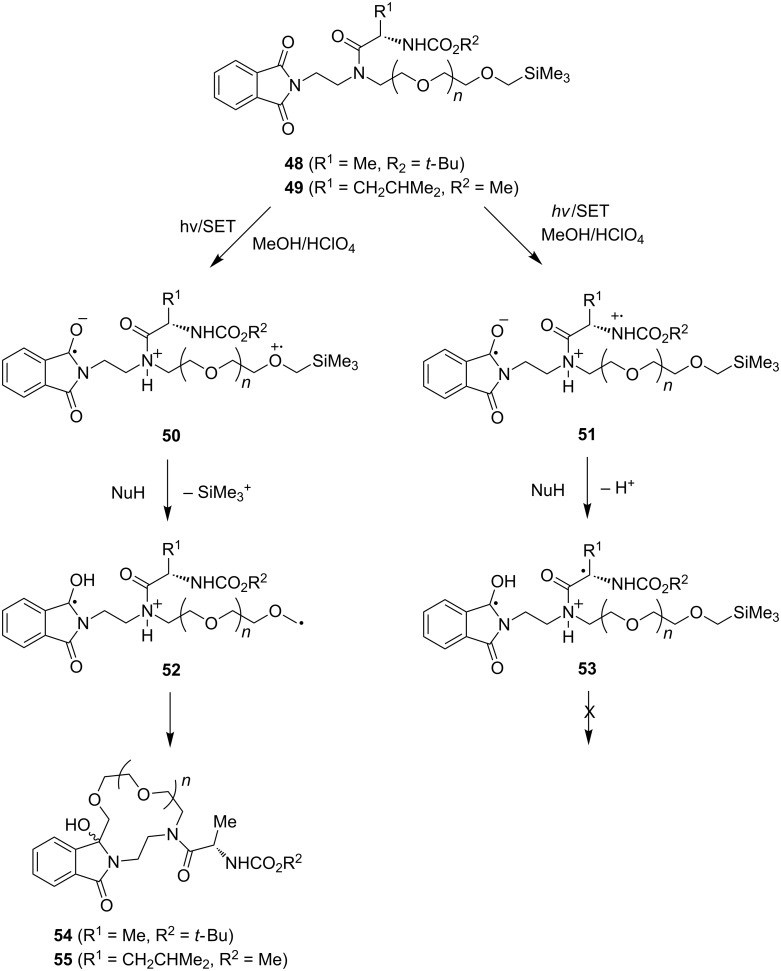
Photoreactions of phthalimide-containing chiral peptide side chains.

Another observation that is related to the synthetic utility of SET-promoted photocyclization reactions driven by chemoselective desilylation of intermediate zwitterionic radicals comes from photochemical investigations of α-silyl ether-terminated phthalimide substrates that contain chiral peptide side chains (Scheme 12) [83]. For example, photoirradiation of acidic methanol solutions containing phthalimides **48** and **49** leads to formation of the respective peptide side chain containing lariat-type azacrown ethers **54** and **55**, each as a mixture of diastereomers. In these processes, SET from the terminal α-silyl ether donor sites to the excited phthlaimide chromophores produces zwitterionic biradicals **50**, which then undergo methanol-assisted desilylation to form biradicals **52**, whose cyclization by C–C bond formation produced the heteroatom-containing, crown ether ring systems. A notable feature of these reactions is their remarkable chemoselectivity. Alternative pathways initiated by intramolecular SET from the carbamate nitrogen donor sites [57] to the phthalimide acceptor moiety excited state, even though thermodynamically possible, do not lead to the generation of cyclic products that would arise by α-CH deprotonation in zwitterionic biradicals **51**.

### SET-promoted photochemical reactions of bis-donor-linked bisphthalimides

**Scheme 13:**
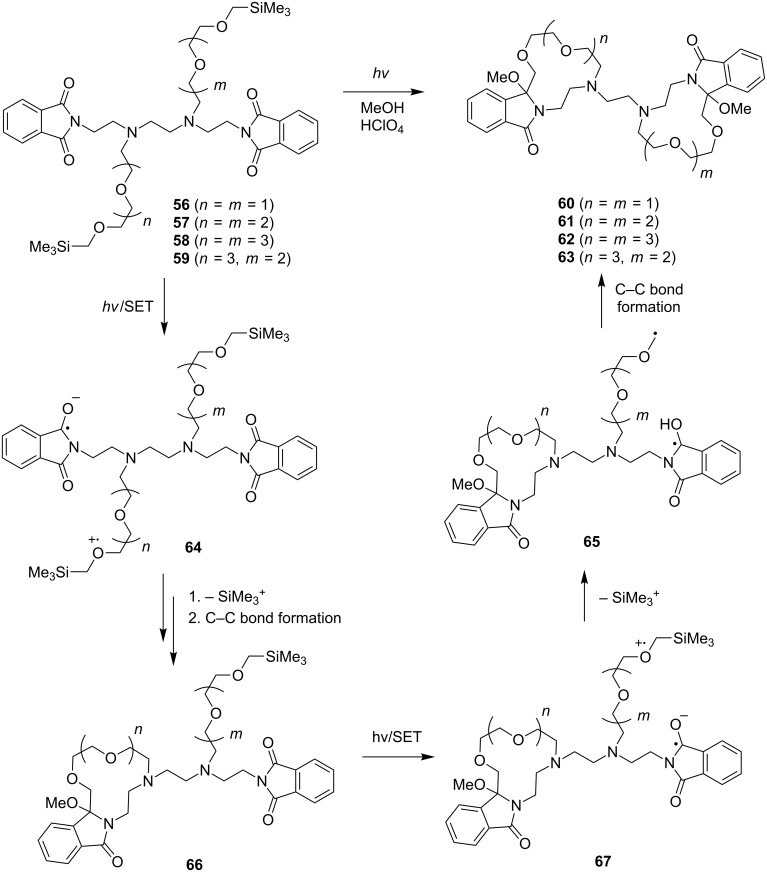
Photoreactions of bis-donor-linked bisphthalimides.

As described above, our studies show that SET-promoted photocyclization reactions of α-silyl ether-terminated, polydonor-linked, imides can be employed as key steps in the synthesis of novel, lariat-type crown ethers. In parallel efforts focusing on bis-donor-linked bis-phthalimides, we have developed a novel strategy for the preparation of bis-crown ethers (Scheme 13) [84]. In these studies, we assumed that photoirradiation of the bis-donor-linked bis-phthalimides could result in the formation of various interconverting zwitterionic biradicals through SET from donor sites in either oxygen-containing chain to either phthalimide acceptor. Based on observations made in earlier studies that the SET process displays a distance-dependence between donor and acceptor, we anticipated that the most efficient reaction pathway would involve a SET between the closest donor and acceptor moieties rather than the more distant pairs and that this event would selectively and sequentially produce zwitterionic biradicals related to **64** and **67** [31–32,67]. Based on this reasoning, we predicted that bis-crown ethers rather than their cross bridged counterparts would be generated in photoreactions of the α-silyl ether-terminated, polyethyleneoxy-linked bis-phthalimides **56**–**59**. Indeed, irradiation of these substances brings about the efficient formation of the respective, novel bis-crown ethers **60**–**63**.

### Indirect approach to the preparation of lariat-type crown ethers

**Scheme 14:**
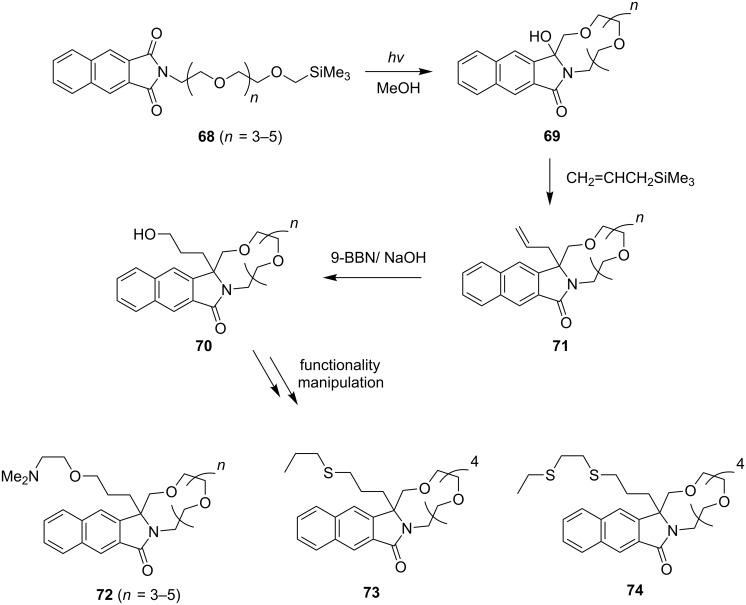
Indirect approach to the preparation of lariat-type crown ethers.

As discussed above, the indirect approach to the synthesis of lariat-type crown ethers involves a two-phase sequence in which initial construction of the crown ether ring system by using SET promoted photoreactions of linked donor-phathalimide substrates is followed by a second phase involving incorporation of a donor side chain. Investigations aimed at evaluating the utility of this strategy focused on the preparation of members of a group of lariat-type crown ethers that could have potentially interesting metal cation binding properties. As can be seen by inspection of the synthetic sequence shown in Scheme 14, the preparation of lariat-type crown ethers **72**–**74** by employing this strategy began with the synthesis of the α-silyl ether-terminated, polyethylenoxy-linked 2,3-naphthalimides **68**. Photoirradiation of methanol solutions containing these substrates brings about the efficient formation of the macrocyclic-amidols **69** via a sequential SET-desilylation pathway. Introduction of various kinds of n-electron donor-containing side chains into the amidols **69** can be easily achieved by employing Lewis acid-catalyzed reactions with allyltrimethylsilane, which produce the allylation products **71**. Hydroboration–oxidation of the terminal olefin moieties in **71** then forms the corresponding alcohols **70** that contain key hydroxypropyl side chains to which various kinds of donor functionality can be appended. By employing this approach, a number of lariat-type azacrown ethers including **72**–**74** which possess aminoether and thioether side chains, have been prepared.

### Lariat-type crown ether-based fluorescence sensors for heavy metal ions

**Scheme 15:**
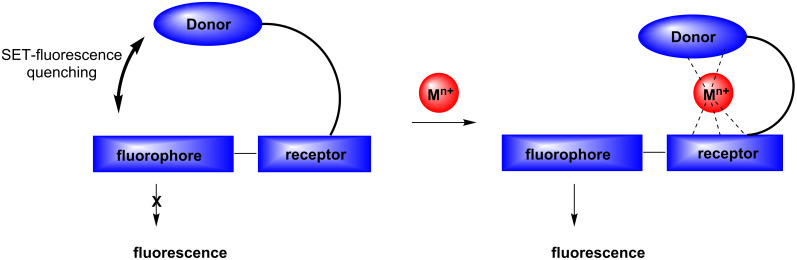
SET-based fluorescence sensing modes according to guest binding.

**Scheme 16:**
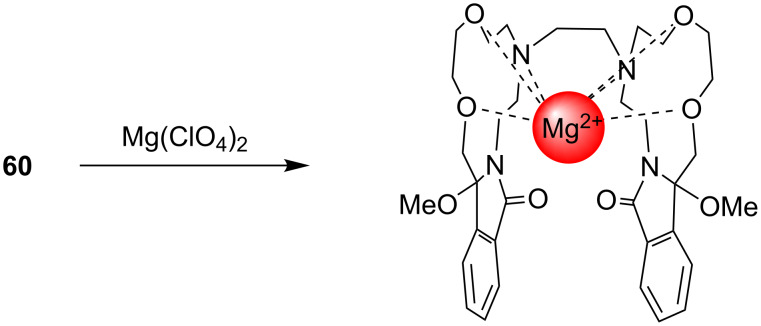
Enhancement of the exciplex formation and fluorescence of bis-crown ether **60** with a Mg^2+^ sandwich-type binding.

Since the pioneering work of Sousa and coworkers [85–86] in the 1970s, intensive and wide-ranging efforts have been dedicated to the development of crown ether based, fluorescence sensing substances for the detection of metal cations. One goal of our investigations was to show that the synthetic strategies based on SET-promoted photocyclization reactions of donor–acceptor-linked substrates could be employed in practical and efficient routes for the preparation of new metal cation-fluorescence sensors. As described earlier by de Silva and others [74–79], SET based fluorescence sensors are useful substances that signal guest-binding by interrupting the SET-quenching of the excited states of the fluorophore moieties that are appended to the host (Scheme 15). We expected that metal cation binding to **72**–**74** would be assisted by the oxygen, nitrogen, and sulfur donors within the pendant side chain. As a consequence, it would be accompanied concomitantly by a dislocation of the side chain moiety and a reduction in intramolecular SET quenching of the singlet excited naphthalene fluorophore by the strong electron donating sulfur and nitrogen atoms. In the studies, it was observed that fluorescence of these and related materials is enhanced in the presence of various kinds of heavy metal cations (in some cases selectively) including Mg^2+^, Cu^2+^, Hg^2+^, and Pb^2+^, as well as Ag^+^ [87–88]. Also, the bis-crown ether **60** forms a complex with metal cations (e.g., Mg^2+^) [84], and the sandwich-type binding of the cation results in a close facial approach of the benzamide aromatic rings in **60**, which results in intramolecular exciplex formation (Scheme 16).

## Conclusion

Direct and indirect approaches, which rely on the use of SET-promoted photocyclization reactions of α-silyl ether-terminated, polydonor-linked, imides (e.g., phthalimides and naphthalimides), have been developed for the synthesis of novel lariat-type crown ethers. Parallel investigations on the reaction mechanisms have provided a useful guideline for predicting the chemo- and regioselectivties and efficiencies of these excited state reactions. These observations demonstrate the unique features of SET-promoted photocyclization reactions and how they can be utilized in routes for the preparation of useful members of the crown ether family including lariat-type crown ethers. It should be noted that, the utility of photochemical reactions in organic synthesis is often limited by scale up issues. However, several features of the currently described SET promoted excited state reactions make them suitable for applications in unique situations, especially when the processes require a high degree of temporal and spatial control.
